# Comparison of a Target Trial Emulation Framework vs Cox Regression to Estimate the Association of Corticosteroids With COVID-19 Mortality

**DOI:** 10.1001/jamanetworkopen.2022.34425

**Published:** 2022-10-03

**Authors:** Katherine L. Hoffman, Edward J. Schenck, Michael J. Satlin, William Whalen, Di Pan, Nicholas Williams, Iván Díaz

**Affiliations:** 1 Division of Biostatistics, Department of Population Health Sciences, Weill Cornell Medicine, New York, New York; 2 Division of Pulmonary and Critical Care, Department of Medicine, Weill Cornell Medicine, New York, New York; 3 Division of Infectious Disease, Department of Medicine, Weill Cornell Medicine, New York, New York; 4 Mailman School of Public Health, Department of Epidemiology, Columbia University, New York, New York

## Abstract

**Importance:**

Communication and adoption of modern study design and analytical techniques is of high importance for the improvement of clinical research from observational data.

**Objective:**

To compare a modern method for statistical inference, including a target trial emulation framework and doubly robust estimation, with approaches common in the clinical literature, such as Cox proportional hazards models.

**Design, Setting, and Participants:**

This retrospective cohort study used longitudinal electronic health record data for outcomes at 28-days from time of hospitalization within a multicenter New York, New York, hospital system. Participants included adult patients hospitalized between March 1 and May 15, 2020, with COVID-19 and not receiving corticosteroids for chronic use. Data were analyzed from October 2021 to March 2022.

**Exposures:**

Corticosteroid exposure was defined as more than 0.5 mg/kg methylprednisolone equivalent in a 24-hour period. For target trial emulation, exposures were corticosteroids for 6 days if and when a patient met criteria for severe hypoxia vs no corticosteroids. For approaches common in clinical literature, treatment definitions used for variables in Cox regression models varied by study design (no time frame, 1 day, and 5 days from time of severe hypoxia).

**Main Outcomes and Measures:**

The main outcome was 28-day mortality from time of hospitalization. The association of corticosteroids with mortality for patients with moderate to severe COVID-19 was assessed using the World Health Organization (WHO) meta-analysis of corticosteroid randomized clinical trials as a benchmark.

**Results:**

A total of 3298 patients (median [IQR] age, 65 [53-77] years; 1970 [60%] men) were assessed, including 423 patients who received corticosteroids at any point during hospitalization and 699 patients who died within 28 days of hospitalization. Target trial emulation analysis found corticosteroids were associated with a reduced 28-day mortality rate, from 32.2%; (95% CI, 30.9%-33.5%) to 25.7% (95% CI, 24.5%-26.9%). This estimate is qualitatively identical to the WHO meta-analysis odds ratio of 0.66 (95% CI, 0.53-0.82). Hazard ratios using methods comparable with current corticosteroid research range in size and direction, from 0.50 (95% CI, 0.41-0.62) to 1.08 (95% CI, 0.80-1.47).

**Conclusions and Relevance:**

These findings suggest that clinical research based on observational data can be used to estimate findings similar to those from randomized clinical trials; however, the correctness of these estimates requires designing the study and analyzing the data based on principles that are different from the current standard in clinical research.

## Key Points

**Question:**

How do modern methods for statistical inference compare with approaches common in the clinical literature when estimating the association of corticosteroids with mortality for patients with moderate to severe COVID-19?

**Findings:**

In a cohort study using retrospective data for 3298 hospitalized patients with COVID-19, target trial emulation using a doubly robust estimation procedure successfully recovers a benchmark from a meta-analysis of randomized clinical trials . In contrast, analytic approaches common in the clinical research literature generally cannot recover the benchmark.

**Meaning:**

These findings suggest that clinical research based on observational data can be used to estimate findings similar to those from randomized clinical trials; however, the correctness of these estimates requires designing and analyzing the data set on principles that are different from the current standard in clinical research.

## Teaser

This cohort study compares methods for statistical inference using observational data in replicating findings from a meta-analysis of randomized clinical trials.

## Introduction

Observational databases are invaluable resources when randomized clinical trials (RCTs) are infeasible or unavailable. However, the correctness of the conclusions gleaned from analyses of observational data hinges on the careful consideration of study design principles and choice of estimation methods.^1,2,3,4^

In this study, we contrast the use of target trial emulation with various traditional analytical approaches using Cox regression. While most epidemiologists and statisticians agree on the importance of a well-defined exposure, outcome, and population of interest, the 2 strategies we compare differ significantly in the subsequent steps to choose a research question and data analysis method.

In the traditional approach to clinical research, the analysis proceeds by postulating a regression model according to the type of data available. For example, when faced with a time-to-event outcome, researchers automatically fit a Cox regression model (often due to limitations in knowledge, time, or software capabilities). The coefficients of the regression model are then used to answer to the clinical question of interest. We refer to this approach as a “model-first” approach, due to the primacy of the regression model.

A model-first approach induces multiple problems for the estimation of effects.^5^ First, regression coefficients often do not represent quantities of primary scientific interest or well-defined effects.^6^ Second, assumptions, such as the proportional hazards assumption used in Cox models, are rarely correct in medical research, since hazards cannot be proportional when a treatment effect changes over time.^7^ Third, regression models cannot correctly handle time-dependent feedback among confounders, treatment, and the outcome.^1,8^ Fourth, the model-first approach yields a tendency to interpret all coefficients in the model, a problem known as the *table 2 fallacy*.^9^ Lastly, model-first approaches fail to account for the variance induced during model selection, thereby leading to incorrect statistical conclusions.^10^

Recent developments in the statistical inference literature provide researchers with a number of tools to alleviate the aforementioned biases. Newer frameworks, such as the target trial emulation^11^ and roadmap for causal inference,^12^ allow researchers to proceed with a question-first approach. Instead of defaulting to estimates provided by regression models, a question-first approach begins by defining a hypothetical target trial and subsequent target of inference that answers the scientific question of interest. This is the so-called *estimand*, or quantity to be estimated. After the estimand is chosen, researchers have the freedom to select an estimation technique that mitigates model misspecification biases. Incorporating these principles can help clarify the research question, determine study eligibility requirements, identify enrollment and follow-up times, decide whether sufficient confounder data are available, and increase the likelihood of obtaining a correct estimate.^13,14^

In this study, we compare a question-first approach against multiple model-first approaches for statistical inference. Our case study is the association of corticosteroids with mortality for patients with moderate to severe COVID-19 using a retrospective cohort of patients at NewYork–Presbyterian Hospital (NYPH) during Spring 2020, at the beginning of the COVID-19 pandemic. Lack of guidance for clinical practice during this period meant that high variability existed in the administration and timing of corticosteroids (eFigure 1 in the Supplement). Clinician practice variability aids in the estimation of treatment effects by yielding data sets with adequate natural experimentation, but the resulting complex longitudinal treatment patterns complicate study design and analytical methods. This observational data set, together with results from numerous RCTs on corticosteroids, provide a unique opportunity to benchmark design and analysis methods. We benchmark our target trial emulation results against outcome measures obtained in the World Health Organization (WHO) RCT meta-analysis.^15^

## Methods

This cohort study was approved by the institutional review board at Weill Cornell Medicine with a waiver of informed consent because it was a minimal risk study that could not be carried out without a waiver of consent due to the retrospective nature and large sample size. The study was designed in April 2020, prior to the results of corticosteroid RCTs and resulting clinical guidance. This report follows the Strengthening the Reporting of Observational Studies in Epidemiology (STROBE) reporting guideline. The question we examined was what is the association of a treatment regimen of corticosteroids administered under the clinical indication of severe hypoxia with mortality for patients hospitalized with COVID-19?

### Hypothetical Target Trial

#### Population

Inclusion criteria were adult patients with SARS-CoV-2 infection who were admitted to NYPH’s Cornell, Lower Manhattan, or Queens locations. SARS-CoV-2 infection was confirmed through reverse-transcriptase–polymerase chain-reaction assay performed on nasopharyngeal swab specimens. The tests were obtained at hospital admission, ie, at the same time of eligibility and time zero. Patients who had chronic use of corticosteroids prior to hospitalization or who were transferred into NYPH from an outside hospital were excluded.

#### Hypothetical Treatment Regimen

In the hypothetical treatment regimen, patients would be randomized on their first day of hospitalization to receive either standard of care therapy (without corticosteroids) or standard of care plus a corticosteroid regimen to be administered if and when criteria for severe hypoxia were met. The corticosteroid dosage was a minimum of 0.5 mg/kg body weight of methylprednisolone equivalent per 24-hour period, and the duration of therapy was 6 days.^16^ Corticosteroids include prednisone, prednisolone, methylprednisolone, hydrocortisone, and dexamethasone, and choice of drug was at the attending physician’s discretion. Severe hypoxia was defined as the initiation of high-flow nasal cannula, venturi-mask, noninvasive or invasive mechanical ventilation, or an oxygen saturation of less than 93% after the patient received 6 L of supplemental oxygen via nasal cannula.

#### Outcome and Estimand

The primary outcome was 28-day mortality from time of randomization. The contrast of interest, or estimand, was the 28-day mortality rate difference comparing actual receipt of the 2 treatment regimens (ie, the per-protocol analysis).

#### Data Analysis Plan

A hypothetical trial can assume no loss to follow-up. Under perfect adherence, we would analyze the difference in proportion of patients who experienced the outcome between the 2 treatment regimens.

### Emulation Using Observational Data

#### Data Source and Cohort

The target trial emulation uses retrospective data from patients at NYPH who met the hypothetical trial’s eligibility criteria from March 1 to May 15, 2020. Demographic, comorbidity, and outcome data were manually abstracted by trained medical professionals into a secure REDCap database (Vanderbilt University).^17^ These were supplemented with an internal COVID-19 data repository housing laboratory, procedure, medication, and flowsheet data documented during standard care.^18^ Patient race and ethnicity were determined via manual abstraction of patient responses and categorized as Asian, Black, White, and other (eg, American Indian or Alaskan Native, Pacific Islander, multiracial, or a patient response of some other race) race and Hispanic or Latinx or non-Hispanic or Latinx ethnicity. Race and ethnicity were included in analyses because they are a potential confounder of the association between the exposure and the outcome. Patients were followed for 28 days from hospitalization and lost to follow-up by discharge or transfer to an external hospital system.

#### Treatment Regimens and Measurement

To emulate the target trial corticosteroid treatment regimen, we estimated the effect associated with a hypothetical dynamic treatment regimen,^19^ whereby each patient is administered 6 days of corticosteroids if and when they meet severe hypoxia criteria. This dynamic regimen was contrasted with a static regimen in which patients never receive corticosteroids.

We measured severe hypoxia using vital signs and flowsheet data and define it in the same way as our target trial. We measured corticosteroid exposure using the medication administration record. We computed cumulative milligram per kilogram dosing of corticosteroids over rolling 24-hour windows, and if a patient received more than 0.5 mg/kg methylprednisolone equivalent, they were denoted as having corticosteroids exposure that day.

**Figure 1.**
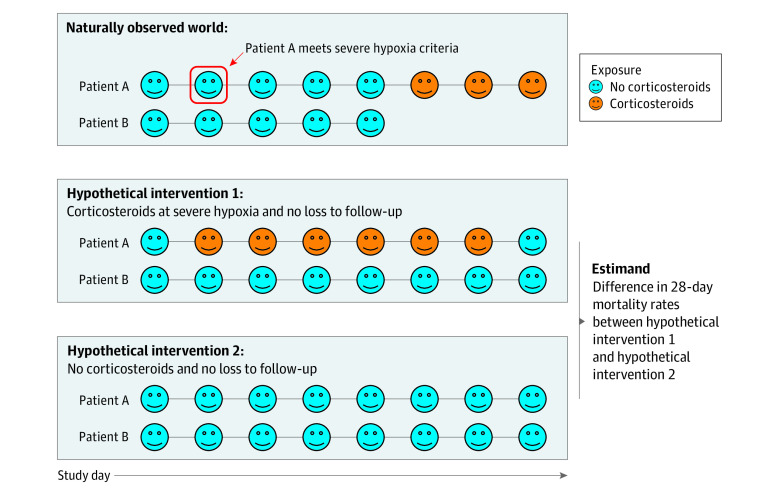
Illustrated Example of 2 Patients Under the 2 Hypothetical Treatment Regimens of the Target Trial Emulation Patient A reached severe hypoxia criteria at study day 2 and was followed the entire study duration. Patient B never reached severe hypoxia criteria and was lost to follow-up after 5 study days. Under the dynamic corticosteroids regimen (intervention 1), patient A received 6 days of corticosteroids, and under intervention 2, they received no corticosteroids. Patient B did not receive corticosteroids under either treatment regimen; however, in both hypothetical worlds, they were observed for the entire study duration.

Since patients in the observed data are subject to loss to follow-up, emulating the trial with observational data requires conceptualizing a hypothetical world where all patients were observed through 28 days. An illustration of the treatment regimens as they related to the observed data are shown in Figure 1.

#### Confounding

In contrast to the hypothetical trial, treatment assignment in the observational study was not randomized and depended on physiological characteristics of each patient. We address confounding in our emulation by adjustment for confounders during analysis. A set of confounders deemed sufficient for adjustment was determined through the expertise of a team of pulmonologists, intensivists, and microbiologists.

**Figure 2.**
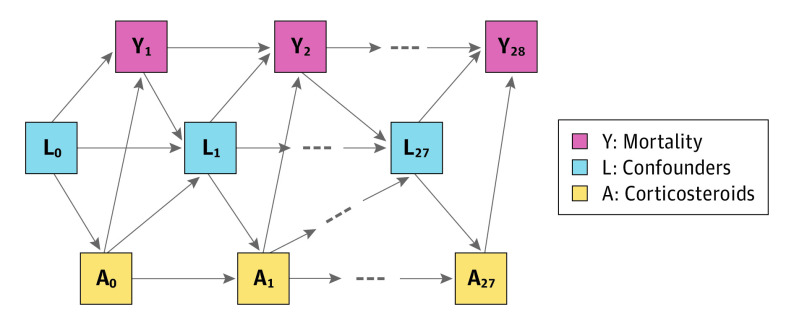
Illustrative Directed Acyclic Graph Showing the Relationship Between Confounders (*L_t_*), Corticosteroid Exposure (*A_t_*), and Mortality (*Y_t_*) Baseline confounders are included in *L*_0_. For simplicity, loss-to-follow-up nodes are not shown. Baseline confounders were age, sex, race, ethnicity, body mass index, comorbidities (coronary artery disease, cerebral vascular event, hypertension, diabetes, cirrhosis, chronic obstructive pulmonary disease, active cancer, asthma, interstitial lung disease, chronic kidney disease, immunosuppression, HIV, and home oxygen use), mode of respiratory support within 3 hours of hospital admission, and hospital admission location. Time-dependent confounders were heart rate, pulse oximetry percentage, respiratory rate, temperature, systolic and diastolic blood pressure, serum urea nitrogen-creatinine ratio, creatinine, neutrophils, lymphocytes, platelets, bilirubin, blood glucose, D-dimers, C-reactive protein, activated partial thromboplastin time, prothrombin time, arterial partial pressure of oxygen, arterial partial pressure of carbon dioxide, mode of respiratory support, vasopressors, diuretics, angiotensin converting enzyme inhibitors/angiotensin-receptor blockers, hydroxychloroquine, and tocilizumab.

Baseline confounders included sociodemographic characteristics, body mass index (BMI), comorbidities, and hospital admission location. Time-dependent confounders included vital signs, laboratory results, cotreatments, and mode of respiratory support. The measurement process (ie, whether a clinician decided to measure these variables) was also an important confounder included in the analysis. Details of confounders are provided in the eMethods in the Supplement. Figure 2 summarizes the relationship between confounders, treatment, and outcomes.

#### Outcome and Estimand

Our estimand of interest is the difference in 28-day mortality rates in a hypothetical world where we had implemented the 2 different corticosteroid treatment regimens, as well as an intervention to prevent loss to follow-up. Under the assumption that treatment and loss to follow-up each day were randomized conditional on the baseline and time-dependent confounders, this estimand is identifiable by a longitudinal *g*-computation formula.^20^ It is important to emphasize that conditional randomization is a key assumption without which the target emulation may fail. This *g*-formula will be our estimand of interest, but we note that it is not the only possible identification strategy (eMethods in the Supplement).

### Statistical Analysis

#### Target Trial Emulation

When using the *g*-formula to estimate effects, correct emulation of a target trial requires proper adjustment for measured confounding. It is important to use estimation methods capable of fitting the data using flexible mathematical relationships so that confounding is appropriately removed, especially when the number of baseline and time-dependent confounders is large.

Methods to estimate the *g*-computation formula (eg, inverse probability weighting, parametric *g*-formula, targeted minimum loss–based estimators, sequentially doubly robust estimators [SDR])^21,22^ rely on 2 kinds of mathematical models: the outcome as a function of the time-dependent confounders and treatment as a function of time-dependent confounders. Methods that use only one of these models are often called *singly robust*, because their correctness relies on the ability to correctly specify one of the models (eg, inverse probability weighting relies on estimating treatment models correctly). Methods that use both of these models are often called *doubly robust*, because they remain correct under misspecification of one of the 2 models.

Furthermore, doubly robust estimators, such as targeted minimum loss–based estimators and SDR, allow the use of machine learning to flexibly fit relevant treatment and outcome regressions.^23,24^ This is desirable because these regression functions might include complex associations, and capturing those associations is not possible using simpler regression such as the Cox model.^25^

The primary analysis is conducted using SDR estimation with a dynamic intervention, time-varying confounders, and a time-to-event outcome. An ensemble of machine learning models using the super learner algorithm is used to estimate the regressions for treatment and outcome.^26,27^ Additional details, including sensitivity analyses, an illustrated analytical file, and code tutorial, are available in eFigure 2 and the eMethods in the Supplement.

#### Model-First Approaches

For contrast with the target trial emulation strategy, we review methods of studies cited in a COVID-19 corticosteroids meta-analysis by Ebrahimi Chaharom et al^28^ and then analyze the data using study designs common in those studies. The data source and outcome are the same as the target trial.

##### Point-Treatment Cox Models

The first approach we explore is a regression for mortality with a point-treatment variable. The inclusion criteria and time zero are defined as the time of meeting hypoxia criteria, which is the intended indication for corticosteroids. A study design using this approach entails several choices, including defining a range of time relative to inclusion criteria for a patient to be considered treated. Once this range is determined, researchers must decide how to handle patients treated before the inclusion time begins or after the treatment interval ends, as well as those who experience the outcome within the treatment interval.

We fit Cox models using data sets obtained from various design choices, summarized in Table 1. Baseline confounders and time-dependent confounders from day zero are included as adjustment variables. The exponentiated coefficient for corticosteroids is interpreted as the hazard ratio (HR) for corticosteroid exposure within the defined treatment window for patients with moderate to severe COVID-19.

**Table 1.** Study Design Specifications for the Model-First Approaches

Model	Study design
A	Corticosteroid exposure was defined as anytime during the course of hospitalization. All patients satisfying inclusion criteria were included in the analysis, and time to event was defined as time from hypoxia to death.
B	Corticosteroid exposure was defined as any administration up to 1 d after meeting hypoxia criteria. All patients satisfying inclusion criteria were included in the analysis, and time to event was defined as time from hypoxia to death.
C	Corticosteroid exposure was defined as any administration up to 1 d after meeting hypoxia criteria. Patients who died during this time window were excluded. Patients who received corticosteroids after the time window were included in the control group.
D	Corticosteroid exposure was defined as any administration up to 1 d after meeting hypoxia criteria. Patients who died during this time window were excluded. Patients who received corticosteroids before hypoxia were excluded. Patients who received corticosteroids after the time window were included in the control group.
E	Corticosteroid exposure was defined as any administration up to 1 d after meeting hypoxia criteria. Patients who received corticosteroids before hypoxia were excluded. Patients who received corticosteroids after the 1-d time window passes were censored at the time of corticosteroids receipt.
F	Corticosteroid exposure was defined as any administration up to 5 d after meeting hypoxia criteria. All patients satisfying inclusion criteria were included in the analysis, and time to event was defined as time from hypoxia to death.
G	Corticosteroid exposure was defined as any administration up to 5 d after meeting hypoxia criteria. Patients who died during this time window were excluded. Patients who received corticosteroids after the time window were included in the control group.
H	Corticosteroid exposure was defined as any administration up to 5 d after meeting hypoxia criteria. Patients who died during this time window were excluded. Patients who received corticosteroids before hypoxia were excluded. Patients who received corticosteroids after the time window were included in the control group.
I	Corticosteroid exposure was defined as any administration up to 5 d after meeting hypoxia criteria. Patients who received corticosteroids before hypoxia are excluded. Patients who received corticosteroids after the 1-d time window passes were censored at the time of corticosteroids receipt.
J	Corticosteroid exposure was allowed to be a time-varying covariate beginning at the time of hospitalization.

These point-treatment estimates apply only to the hypoxic population. They are different from the estimates in the target trial emulation, which apply to the population of hospitalized patients. These estimations are the closest possible analog we can obtain within a model-first framework using a point-treatment.

##### Time-Varying Cox Models

In the second model-first approach, we fit a time-varying Cox model for time to mortality up to 28 days from the day of hospitalization. This model uses the entire cohort and contains baseline and time-dependent confounders, as well as daily corticosteroid administration. The coefficient for corticosteroids is exponentiated and used as an estimate of the HR for mortality associated with corticosteroids in hospitalized patients with COVID-19.

#### RCT Meta-analysis Benchmark

Several RCTs have established the effectiveness of corticosteroids in the treatment of patients with moderate to severe COVID-19.^29,30,31^ The WHO performed a meta-analysis of 7 such RCTs and estimated the odds ratio (OR) for the association of corticosteroids with mortality to be 0.66 (95% CI, 0.53-0.82).^15^ We use this estimate, as well as supporting evidence from other RCT meta-analyses^28,32^ to benchmark our results. A discussion of assumptions for benchmarking, along with comparisons of our target trial study design, population, and treatment arms to the benchmark RCTs, are provided in eTable 1, eTable 2, and the eAppendix in the Supplement.

## Results

### Target Trial Emulation

In the target trial emulation analysis, all 3298 patients (median [IQR] age, 65 [53-77] years; 1970 [60%] men) who were admitted to the hospital were analyzed. Table 2 and eTable 4 in the Supplement display characteristics of the cohort, and eTable 5 in the Supplement describes the informative measurement process. There were 1690 patients who reached severe hypoxia and 423 patients who received corticosteroids at any point during follow-up; 699 patients died before 28 days.

**Table 2.** Demographic Characteristics and Outcome for Study Cohort Overall and Stratified by Any Corticosteroid Exposure

Characteristic^a^	No. (%)		
	Overall (N = 3298)	Corticosteroids	
		Never (n = 2875)	Ever (n = 423)
Age, median (IQR), y	65 (53-77)	65 (52-77)	67 (58-75)
Sex			
Women	1328 (40)	1178 (41)	150 (35)
Men	1970 (60)	1697 (59)	273 (65)
Race			
Asian	602 (18)	517 (18)	85 (20)
Black	399 (12)	352 (12)	47 (11)
White	938 (28)	818 (28)	120 (28)
Other^a^	1141 (35)	1009 (35)	132 (31)
Unknown or declined	218 (7)	179 (6)	39 (9)
Ethnicity			
Hispanic or Latinx	1117 (34)	994 (35)	123 (29)
Non-Hispanic or Latinx	1585 (48)	1388 (48)	197 (47)
Unknown or declined	596 (18)	493 (17)	103 (24)
BMI, median (IQR)^b^	27 (23-31)	27 (23-31)	28 (24-32)
Home supplemental oxygen	312 (10)	286 (10)	26 (6)
Coronary artery disease	460 (14)	402 (14)	58 (14)
Diabetes	1033 (31)	891 (31)	142 (34)
Hypertension	1780 (54)	1544 (54)	236 (56)
Cerebral vascular event	225 (7)	193 (7)	32 (8)
Cirrhosis	35 (1)	30 (1)	5 (1)
CKD/ESKD	159 (5)	146 (5)	13 (3)
Asthma	180 (6)	145 (5)	35 (8)
COPD	134 (4)	100 (4)	34 (8)
Active cancer	136 (4)	118 (4)	18 (4.3)
Immunosuppressed	51 (2)	44 (2)	7 (1.7)
ILD	5 (<1)	3 (<1)	2 (1)
HIV	35 (1)	33 (1)	2 (1)
Active smoker	104 (3)	93 (3)	11 (2.6)
Former smoker	543 (16)	442 (15)	101 (24)
Outcome: 28-d mortality	699 (21)	574 (20)	125 (30)

Abbreviations: BMI, body mass index (calculated as weight in kilograms divided by height in meters squared); CKD, chronic kidney disease; COPD, chronic obstructive pulmonary disease; ESKD, end stage kidney disease; ILD, interstitial lung disease.

^a^ Other race category includes American Indian or Alaskan Native, Pacific Islander, multiracial, or a patient response of some other race.

^b^ 190 patients (5.8%) did not have BMI data available.

The estimated mortality rate under the no corticosteroids regimen was 32.2% (95% CI, 30.9%-33.5%). The estimated mortality rate under the corticosteroids regimen was 25.7% (95% CI, 24.5%-26.9%). This yields an estimated mortality reduction of 6.5% (95% CI, 5.7%-7.4%) if this policy had been implemented. Sensitivity analyses yield near-identical results (eAppendix in the Supplement).

### Model-First Approaches

In the subset of patients who met severe hypoxia criteria, 72 patients received corticosteroids within 1 day of hypoxia and 191 patients received corticosteroids within 5 days of hypoxia. There were 18 patients who died within 1 day of hypoxia without receiving corticosteroids and 451 patients who died within 5 days of hypoxia without receiving corticosteroids.

**Figure 3.**
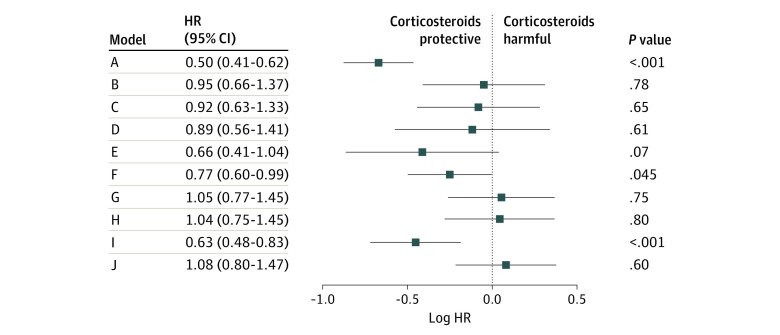
Forest Plot of Model-First Results Study designs A through J are described in Table 1. HR indicates hazard ratio.

Model A, which defined corticosteroid exposure as anytime during hospitalization, yielded an HR of 0.50 (95% CI, 0.41-0.62). Models B through I, which placed either a 1- or 5-day limit on corticosteroids treatment from the time of hypoxia, mostly did not yield statistically significant results in either direction (model B: HR, 0.95 [95% CI, 0.66-1.37]; model C: HR, 0.92 [95% CI, 0.63-1.33]; model D: HR, 0.89 [95% CI, 0.56-1.41]; model E: HR, 0.66 [95% CI, 0.41-1.04]; model G: HR, 1.05 [95% CI, 0.77-1.45]; model H: HR, 1.04 [95% CI, 0.75-1.45]). The exception to this was model I, which excluded patients who died before 5 days and estimated the HR to be 0.63 (95% CI, 0.48-0.83). Model F also reached statistical significance (HR, 0.77 [95% CI, 0.60-0.99]) and was the result of a 5-day treatment window with no exclusion or censoring variations. The time-varying Cox model yielded an HR of 1.08 (95% CI, 0.80-1.47). Figure 3 summarizes the model-first results.

## Discussion

This cohort study illustrates how a question-first approach can aid in devising an optimal design and choice of estimation procedure for an analysis of observational data. We show that using the target trial framework succeeds in recovering the benchmark findings obtained in a meta-analysis of RCTs. Our estimate that corticosteroids would be associated with reduced overall 28-day mortality in a hospitalized cohort is equivalent to an OR of 0.73 (95% CI, 0.68-0.74), which is qualitatively the same as the WHO’s OR estimate of 0.66 (95% CI, 0.53-0.82). Our study design allowed us to conceptualize a meaningful intervention, ie, randomize patients at hospitalization but do not give corticosteroids unless the patient becomes severely hypoxic. Our analysis plan enabled us to flexibly adjust for a large number of potential time-dependent confounders.

In contrast, most model-first approaches could not recover the RCT benchmark using the same data source. This finding aligns with other corticosteroids research. A meta-analysis by Ebrahimi Chaharom et al^28^ containing observational analyses for more than 18 000 patients found no overall association of corticosteroid use with mortality (OR, 1.12 [95% CI, 0.83-1.50]).^28^ The task of creating reliable evidence from complex longitudinal data is not an easy one, and many of these studies have flaws in their designs.

We found most studies in the current observational corticosteroids literature allowed the treated group to receive corticosteroids anytime during hospitalization.^33,34,35^ This is problematic because it introduces an immortal time bias, which biases results toward a protective association of corticosteroids.^36^ A few studies did limit the treatment time frame in an effort to diminish immortal time bias. The grace period for treatment was handled in various ways, eg, excluding patients who died prior to a time window after inclusion criteria,^35,37,38^ or excluding patients who received treatment after the treatment window ended.^39^ Both exclusions may lead to bias and spurious associations.^1^ An alternative to exclusion is censoring patients at their time of receiving treatment if that time is after the treatment window passes; however, Cox regression cannot handle time-dependent censoring.^1,8^

In addition to these issues, it is often unclear in the literature how patients who received corticosteroids prior to meeting inclusion criteria are handled in the analysis.^39,40^ A related issue is that corticosteroids can affect severity of illness. All of the point-treatment studies are thus subject to collider bias by subsetting to patients who are severely ill.^41^ While the time-varying Cox approach does not have the same time-alignment biases as the point-treatment design, it cannot properly account for time-dependent confounders.^1^ Additionally, much of observational research on corticosteroids uses propensity score matching, reweighting, or model selection (eg, stepwise regression). However, no estimation method can solve these study design issues,^1^ and inappropriate model selection induces problems in computation of SEs.^10^ These biases appear in our model-first results; the study designs that found a statistically significant protective association of corticosteroids had extreme immortal time bias through undefined or extended treatment time windows (models A, F, and I).

### Limitations

This study has some limitations. First, while the study’s time frame before publication of the results of any RCTS on the efficacy of corticosteroids against COVID-19 is ideal for natural experimentation and the estimation of outcomes, it includes surge conditions and rapidly changing clinical practice, challenging the assumptions needed for transportability and benchmarking. Second, we cannot rule out unmeasured confounding in the treatment, censoring, or outcome mechanisms. Specifically, the different discharge pathways (eg, home, nursing home) may be associated with unmeasured patient characteristics and lead to very different outcomes. Third, we did not have the data to look at individual corticosteroid types, making comparisons to a specific RCT impossible. Fourth, the binning of our data into 24-hour intervals may induce issues related to the correct time-ordering of events.

## Conclusions

The findings of this cohort study may serve as an example in which the current standard for clinical research methods fails to estimate the correct treatment outcome where a target trial emulation method succeeds. Using observational data to guide clinical practice is possible but relies on the use of contemporary statistical and epidemiological principles. We hope this study and accompanying technical guide encourages adoption of similar innovative techniques into study designs and statistical analyses for observational medical research.

## Notes

Supplement.
eMethods.
**eAppendix.** Supplementary Results **eTable 1.** RECOVERY Study Design Comparison **eTable 2.** RECOVERY Study Population Comparison **eTable 3.** RCT Meta-analysis Corticosteroid Intervention Comparison **eTable 4.** Corticosteroid Types, Cotreatments, and Discharge Pathways **eTable 5.** Informative Measurement Over Time **eFigure 1.** Treatment Timelines for 50 Patients **eFigure 2.** Illustration of Analytical Data Structure
eReferences.
